# Social Media and Body Dissatisfaction: Investigating the Attenuating Role of Positive Parent–Adolescent Relationships

**DOI:** 10.1007/s10964-018-0956-9

**Published:** 2018-11-26

**Authors:** Dian A. de Vries, Helen G. M. Vossen, Paulien van der Kolk – van der Boom

**Affiliations:** 1 Department of Youth and Family, Utrecht University, Heidelberglaan 1, 3584 CS Utrecht, The Netherlands; 2 Department of Child and Adolescent Studies, Utrecht University, Heidelberglaan 1, 3584 CS Utrecht, The Netherlands; 3 Danita Supervised Living, Timon Youth Care, Adriaan Kluitstraat 38, 3052 RD Rotterdam, The Netherlands

**Keywords:** Social networking sites, Adolescence, Body image, Socio-cultural influences, Parenting, Relationships

## Abstract

Previous research has shown that adolescents´ social media use predicts increased body dissatisfaction. However, little is known about social environmental factors that may attenuate this relationship. This study examines one such potential moderating social environmental factor: positive parent–adolescent relationships. A school-based survey was conducted among 440 adolescents aged 12 to 19 (*M=* 14.9, *SD=* 1.8, 47% female). On average, social media use was positively associated with body dissatisfaction, but this relationship was weaker among adolescents who reported a more positive mother–adolescent relationship. Positive father–adolescent relationship did not moderate the association between social media use and body dissatisfaction. These findings may indicate that adolescents’ social environment, notably the relationship they have with their mothers, can protect them against the detrimental effects of social media use on body dissatisfaction. However, longitudinal and experimental studies are needed to examine the direction of effects and test the validity of this interpretation.

## Introduction

Body dissatisfaction, a negative attitude towards the own physical appearance, is one aspect of the broader concept of body image (i.e., the views that individuals have of their physical appearance) (Heider et al. 2018). Body dissatisfaction increases during adolescence and particularly adolescent girls report high levels of body dissatisfaction (Bucchianeri et al. 2013). As body dissatisfaction is detrimental to wellbeing (Stice and Bearman 2001), it is important to identify its correlates. One activity shown to contribute to body dissatisfaction is social media use (Holland and Tiggemann 2016). However, not all adolescents are susceptible to the effects of social media on body dissatisfaction to the same extent. Research has shown that individual factors, such as the tendency to engage in social comparison (Kleemans et al. 2018) and level of media literacy (McLean et al. 2016), moderate social media effects on body dissatisfaction. However, the potential attenuating role of social environmental factors, such as positive parent–adolescent relationships, has not received much attention in research on social media and body image. The current study examines if positive parent–adolescent relationships moderate the association between social media use and body dissatisfaction.

### A Sociocultural Approach to Body Dissatisfaction

A useful framework to study influences on body dissatisfaction is the sociocultural model (Thompson et al. 1999). According to the sociocultural model, adolescents receive messages about what their bodies should look like from different sources, such as their parents, peers, and the media (Thompson et al. 1999). These messages can, for example, include that it is important to be thin or that you should be muscular. If adolescents internalize these appearance ideals as the standards for their own body, they will compare their body against these ideals (Thompson et al. 1999). When their appearance does not match the internalized ideals this will result in body dissatisfaction (Thompson et al. 1999). The validity of the sociocultural model is underscored by a large number of studies. For example, research shows that the pressure to lose weight or gain muscle that adolescents experience from parents, peers, and the media is positively related to their body dissatisfaction (Xu et al. 2010). Furthermore, internalization of appearance ideals and comparison with these ideals was shown to mediate the effects of sociocultural messages about appearance on body dissatisfaction (Shroff and Thompson 2006).

### Social Media and Body Dissatisfaction

Although the sociocultural model originally focused on face-to-face communication and traditional media (e.g., magazines and TV) (Thompson et al. 1999), messages about appearance ideals are now also communicated through social media. On social media, adolescents post photographs of themselves and view photos of others (Espinoza and Juvonen 2011). Physical appearance plays an important role in these activities (Siibak 2009). Adolescents report that they experience pressure to “look perfect” on social media and carefully select and edit their posts to do so (Chua and Chang 2016). Moreover, adolescent boys and girls who spend more time on social media receive more feedback about their appearance (de Vries et al. 2016). In addition to receiving messages about their own bodies on social media, adolescents see carefully selected and edited pictures of their social media connections (i.e., friends, friends of friends, and celebrities) and the comments they receive. Thus, social media use exposes adolescents to appearance-related messages both in the form of comments about their own body and through viewing what their social media connections find attractive about themselves and others.

In line with the sociocultural model, several studies show that adolescents internalize the appearance ideals conveyed to them through social media and compare themselves with these ideals. For example, in a cross-sectional study adolescents girls who used social media more frequently reported greater internalization of beauty ideals (Vandenbosch and Eggermont 2012). In addition, a longitudinal survey study showed that viewing others’ social media posts was related to social comparison concurrently among adolescent boys and girls and over time among adolescent boys (Rousseau et al. 2017).

As also predicted by the sociocultural model, research shows that the internalization of and comparison with the appearance ideals conveyed through social media results in body dissatisfaction. For instance, social media use was positively related to body dissatisfaction through the internalization of thin ideals and appearance comparison in a cross-sectional survey among adolescent girls (Tiggemann and Miller 2010). Another study found that viewing others’ social media posts was indirectly related to increased body dissatisfaction through social comparison concurrently among adolescent girls and concurrently and longitudinally among adolescent boys (Rousseau et al. 2017). Moreover, an experimental study showed that viewing edited photos of other girls on social media increased body dissatisfaction among adolescent girls, especially those who said they often compared themselves to others (Kleemans et al. 2018). Although most research has focused on girls because girls report higher levels of body dissatisfaction on average, more frequent social media use predicts increased body dissatisfaction among boys and girls to the same extent (de Vries et al. 2016). Overall, the sociocultural model (Thompson et al. 1999) and cross-sectional (Tiggemann and Miller 2010), longitudinal (Rousseau et al. 2017), and experimental (Kleemans et al. 2018) studies thus suggest that social media use is positively related to adolescent boys’ and girls’ body dissatisfaction.

### Parent–Adolescent Relationships and Body Dissatisfaction

Adolescents’ body dissatisfaction is also subject to parental influences (Bearman et al. 2006). Parents not only convey messages about appearance ideals to their children, but the parent–adolescent relationship itself also plays a role in the development of adolescents’ body dissatisfaction (Bearman et al. 2006). Researchers argue that when individuals feel secure in their relationships they are less likely to think that they have to conform to appearance ideals in order to gain others’ acceptance (Holsen et al. 2012). In line with this idea, research has shown that adolescents who experience better parent–adolescent relationships are less dissatisfied with their bodies cross-sectionally (Holsen et al. 2012) and become less dissatisfied with their bodies over time (Bearman et al. 2006). Gender has not been shown to moderate these associations: parent–adolescent relationships contributed to the body dissatisfaction of boys and girls to the same extent (Bearman et al. 2006).

Although gender did not moderate the effects of parent–adolescent relationships on body dissatisfaction when parent–adolescent relationships was measured as a composite of both parents combined (Bearman et al. 2006), there is an indication that relationships between adolescents and their mothers and between adolescents and their fathers may affect daughters and sons differently. For example, experiencing fewer difficulties in talking with their fathers was related to lower body weight dissatisfaction both among male and female adolescents (Al Sabbah et al. 2009). However, difficulties in talking with their mother was only associated with body weight dissatisfaction among girls (Al Sabbah et al. 2009). In another study, maternal intimacy and knowledge were negatively associated and negative maternal conflict was positively associated with increases in weight concern only among adolescent girls (May et al. 2006). However, with respect to father–adolescent relationships, only conflict was positively related to increases in weight concern, both among girls and boys (May et al. 2006). So, although the exact differences are unclear, previous research suggests that mother–adolescent relationships and father–adolescent relationships are differentially associated with adolescents’ body dissatisfaction and this association also depends on the adolescent’s gender.

Aside from the promotive influence of positive parent–adolescent relationships on body image, positive parent–adolescent relationships may also have a protective influence. According to ecological perspectives, social environmental factors (i.e., parents, peers, the community, and the media) not only have unique effects but also shape each other’s influence on developing youth (Jordan 2004). In line with this view, media can have different effects on adolescents depending on their family context (Fikkers et al. 2013). These differential effects of media are attributed to differential processing of media (Fikkers et al. 2016). Specifically to the current topic, positive parent–adolescent relationships may protect adolescents against deleterious sociocultural influences on body dissatisfaction by influencing how they process messages about appearance (Stein and Corte 2003).

Researchers argue that individuals who have good bonds with their parents while they are growing up have a more stable sense of self, which makes their self-worth less dependent on social validation and comparison with others (Stein and Corte 2003). As a result, individuals who experience good relationships with their parents are less likely to internalize and compare themselves with appearance ideals and therefore protected against unwanted sociocultural influences on body dissatisfaction (Cheng and Mallinckrodt 2009). If positive parent–adolescent relationships also result in less internalization of and comparison with the appearance ideals encountered on social media, positive parent–adolescent relationships should attenuate the association between social media use and body dissatisfaction.

## The Current Study

The current cross-sectional survey study examines the associations between social media use, positive parent–adolescent relationships, and body dissatisfaction among adolescents. According to the sociocultural model (Thompson et al. 1999) and previous research (Holland and Tiggemann 2016), adolescents internalize the appearance ideals that are conveyed on social media and compare themselves to these ideals, which results in body dissatisfaction. Therefore, it is hypothesized that adolescents who use social media more frequently will be more dissatisfied with their bodies than adolescents who use social media less frequently (Hypothesis 1).

Previous research also indicates that individuals who have more positive relationships with their parents are less likely to internalize the appearance ideals that they encounter and are therefore less dissatisfied with their bodies (Cheng and Mallinckrodt 2009). In line with previous research (Bearman et al. 2006), it is thus hypothesized that adolescents who have more positive relationships with their mothers and fathers will be less dissatisfied with their bodies (Hypothesis 2). Furthermore, because adolescents who have more positive relationships with their parents process messages about physical appearance in a less negative way (Cheng and Mallinckrodt 2009), they are expected to be less susceptible to the appearance messages on social media. Therefore, it is hypothesized that the positive association between social media use and body dissatisfaction will be attenuated among adolescents who have more positive relationships with their mothers and fathers (Hypothesis 3).

Most research on the relationship between social media use and body dissatisfaction has exclusively focused on females (Holland and Tiggemann 2016). However, in research that included both boys and girls, social media use was positively related to body dissatisfaction to the same extent among both genders (de Vries et al. 2016). Similarly, parent–adolescent relationships were correlated with body dissatisfaction among both boys and girls (Bearman et al. 2006). However, when examined separately, mother–adolescent and father–adolescent relationships may relate to body dissatisfaction of adolescent sons and daughters in different ways (Al Sabbah et al. 2009). This study therefore includes both boys and girls, examines positive mother–adolescent and positive father–adolescent relationships separately, and investigates if gender moderates the associations between body dissatisfaction and a) social media use, b) positive mother–adolescent relationship and positive-father relationship, and c) the interaction between social media use and positive mother–adolescent relationship and positive father–adolescent relationship. Because girls and older adolescents report more body dissatisfaction on average (Bucchianeri et al. 2013), gender and age will be controlled for in all analyses.

## Methods

### Participants and Procedure

The sample was a convenience sample recruited by eight graduate students in 2016. These students recruited adolescents through high schools in the Netherlands. A total of 440 adolescents, aged 12 to 19 years, participated in the study. Of these 440 participants, six did not complete the measure of body dissatisfaction, eight did not report on their social media use, eight did not report on their relationship with their mother, twelve did not report on their relationship with their father, three did not report their gender, and six did not report their age. The mean age of the sample was 14.86 years (*SD* = 1.79). Of the participants who reported their age, 12.0% was twelve (*N=* 52), 18.2% was thirteen (*N=* 79), 11.8% was fourteen (*N=* 51), 13.4% was fifteen (*N=* 58), 22.6% was sixteen (*N=* 98), 18.7% was seventeen (*N=* 81), 3.2% was eighteen (*N=* 14), and 0.2% was 19 (*N=* 1). Gender distribution of the sample was about equal with 232 boys (53.1%) and 205 girls. The distribution of the level of education of the participants was: 50.9% higher level of education, 33.9% middle level of education, and 15.1% lower level of education. Concerning ethnicity, 87.6% of the mothers and 87.2% of the fathers of the adolescents in this sample were born in the Netherlands. This means that the majority of adolescents did not have, what in the Netherlands is referred to as a “migration background.” In the general Dutch population 19.6% of fourteen-year-olds has a migration background (Statline 2018). Therefore, the sample is somewhat less ethnically diverse than the average Dutch population of adolescents.

Before the adolescents participated in the study, their parents were informed about the purpose and procedure of the study and given the opportunity to retract participation of their child. Adolescents were also informed about the purpose and procedure of the study and provided active informed consent. After consent was provided, adolescents completed a paper-pencil questionnaire that took approximately 30 min to fill out.

### Measures

#### Body dissatisfaction

The body dissatisfaction subscale of the Body Attitude Test (BAT; Probst et al. 1995) was used to measure body dissatisfaction. This subscale consists of four items with a seven-point Likert scale ranging from *completely disagree* (1) to *completely agree* (7). An example item is: “When I compare my body to that of my peers, I am dissatisfied with my body.” The reliability of this subscale was good (α = .87). A mean score was calculated for which higher scores indicted greater body dissatisfaction (*M* = 2.81, *SD* = 1.43).

#### Social media use

An adapted version of the Multidimensional Scale of Facebook Use (MSFU; Frison and Eggermont 2016) was used to measure social media use. The MSFU asks about three types of Facebook use: passive Facebook use (e.g., “How often do you visit the profile of a Facebook friend?”), active private Facebook use (e.g., “How often do you send a private message to a Facebook friend?”) and active public Facebook use (e.g., “How often do you post a message on your Facebook timeline?”) Items were rephrased so they would not specifically refer to Facebook but to social network sites in general (e.g., also Instagram). For example, the item “How often do you post a photo on your own Facebook timeline” was rephrased to “How often do you post a photo on social network sites.” Originally the MSFU has seven response options: (1) *never*, (2) *less than once a month*, (3) *one to three times a month*, (4) *once per week*, (5) *multiple times a week*, (6) *daily*, and (7) *multiple times a day*. Because recent statistics show that adolescents often use social media constantly throughout the day (Wennekers et al. 2016) an additional category: (8) *all day long* was included. A total social media score was calculated by averaging all items. A higher score indicated more frequent social media use (*M* = 3.92, *SD* = 1.41, α = 0.88).

#### Positive parent–adolescent relationships

The Network of Relationships Questionnaire – Relationship Qualities Version (NRI-RQV; Buhrmester and Furman 2008), was used to measure positive parent–adolescent relationships. This questionnaire consists of ten subscales: five measuring positive qualities of the parent–adolescent relationship (i.e., companionship, intimate disclosure, emotional support, approval, and satisfaction) and five measuring negative qualities of the parent–adolescent relationship (i.e., conflict/quarreling, criticism, pressure, dominance, and exclusion). This study only includes the positive subscales. Participants indicated how often the positive qualities occurred on a five-point scale ranging from *never or hardly at all* (1) to *always or extremely much* (5). All items were asked for mother and father separately. A mean score was calculated to represent a positive father–adolescent relationship measure (*M* = 3.40, *SD* = 0.64, α = 0.91) and a positive mother–adolescent relationship measure (*M* = 3.72, *SD* = 0.63, α = 0. 92). Higher scores on these composites indicated more positive parent–adolescent relationships.

### Statistical Analyses

First, bivariate correlations were calculated to explore the relation between all model variables. However, as these correlations do not control for confounding influences, regression analyses were used to test the hypotheses. A three stage hierarchical multiple regression analysis was conducted with body dissatisfaction as the dependent variable. Model 1 included social media use as well as the covariates age and gender. In Model 2 positive father–adolescent relationship and positive mother–adolescent relationship were added and in Model 3 the interactions between social media use and positive father–adolescent relationship and positive mother–adolescent relationship were added. Predictors were centered prior to computing interaction terms (Cohen et al. 2003). Bootstrap confidence intervals were calculated to deal with the slightly skewed distribution of body dissatisfaction (Desharnais et al. 2015).

## Results

### Descriptive Statistics

Table 1 demonstrates the bivariate correlations between all model variables.

**Table 1:** Bivariate correlations between all model variables

	1	2	3	4	5
1. Body dissatisfaction	1				
2. Social media use	.026^**^	1			
3. Positive father–adolescent relationship	−0.11^*^	0.04	1		
4. Positive mother–adolescent relationship	−0.07	0.08	0.32^**^	1	
5. Gender (0 = boys, 1 = girls)	0.39^**^	0.28^**^	0.05	0.23^**^	1
6. Age	0.20^**^	−0.06	−16^**^	−0.14^**^	−0.02

Note.

*p < 0.05, ** *p* < 0.001

### Social Media Use as a Predictor of Body Dissatisfaction

Model 1, *R*^*2*^ = 0.244, tested the hypothesis that social media use was positively related to body dissatisfaction (H1). The results demonstrate a significant positive relation between social media use and body dissatisfaction, *β=* 0.187, *B=* 0.189, *SE* = 0.045, *p* < 0.001 (Table 2, Model 1). This indicates that adolescents who report more frequent social media use also report higher levels of body dissatisfaction. Therefore, the first hypothesis is supported.

**Table 2:** Hierarchical regression analysis with body dissatisfaction as dependent variable

	Model 1				Model 2				Model 3			
Variable	*B*	*SE*	95% *BCI*	β	*B*	*SE*	95% *BCI*	β	*B*	*SE*	95% *BCI*	β
Age	0.18	0.04	[0.11, 0.29]	0.22***	0.16	0.04	[0.09, 0.23]	0.20***	0.16	0.04	[0.09, 0.23]	0.20***
Gender	1.03	0.13	[0.77, 1.29]	0.36***	1.11	0.14	[0.84, 1.37]	0.39***	1.10	0.13	[0.84, 1.36]	0.39***
Social media use	0.19	0.05	[0.10, 0.29]	0.19***	0.19	0.05	[0.10, 0.29]	0.19***	0.18	0.05	[0.09, 0.28]	0.18***
Positive father–adolescent relationship					−0.13	0.10	[−0.33, 0.06]	−0.06	−0.16	0.09	[−0.36, 0.02]	−0.07
Positive mother–adolescent relationship					−0.30	0.10	[−0.56, −0.02]	−0.13**	−0.27	0.13	[−0.52, −0.01]	−0.12*
Social media use x Positive father–adolescent relationship									0.03	0.07	[−0.12, 0.17]	0.02
Social media use x Positive mother–adolescent relationship									−0.20	0.08	[−0.36, −0.04]	−0.12**
*R*^*2*^		0.24				0.27			0.28			
Δ*R*^*2*^		0.24				0.02			0.01			
*F* for change in *R*^*2*^		44.33***				6.80**			3.74*			

Note.

* *p* < .05, ** *p<* .01, *** *p* < .001

### Positive Parent–Adolescent Relationships as Predictors of Body Dissatisfaction

In Model 2, *R*^*2*^ = 0.268, positive father–adolescent relationship and positive mother–adolescent relationship were added as predictors in order to test whether positive parent–adolescent relationships were associated with lower levels of body dissatisfaction (H2). The results show that adolescents who reported a more positive mother–adolescent relationship experienced lower levels of body dissatisfaction, *β=* −0.132, *B=* −0.297, *SE* = 0.103, *p* = 0.004 (Table 2, Model 2). Positive father–adolescent relationship was unrelated to body dissatisfaction, *β=* −0.059, *B=* −0.131, *SE* = 0.100, *p* = 0.192. Thus, the second hypothesis was partially supported.

### Interaction Between Social Media Use and Positive Parent–Adolescent Relationships

**Fig. 1:**
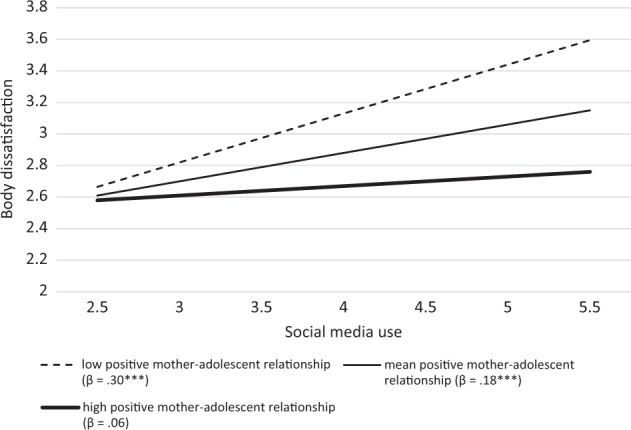
Simple slopes for the relation between social media use and body dissatisfaction calculated at one SD below the mean (low-positive mother–adolescent relationship), the mean (average-positive mother–adolescent relationship), and one SD above the mean (high-positive mother–adolescent relationship) of positive mother–adolescent relationship. Note. Low, mean, and high positive mother–adolescent relationship represent values of respectively 3.08, 3.72, and 4.35. * *p* < 0.05, ** *p<* .01, *** *p* < .001

In Model 3, *R*^*2*^ = 0.281, two interaction terms were included to test whether the relation between social media use and body dissatisfaction was attenuated by positive parent–adolescent relationships (H3). The results (Table 2, Model 3) demonstrate that only the interaction between social media use and positive mother–adolescent relationship was significant, *β=* −0.122, *B=* −0.200, *SE* = 0.076, *p* = 0.009. Figure 1 illustrates that as the level of positive mother–adolescent relationship increases, the relation between social media use and body dissatisfaction becomes weaker. The Johnson-Neyman technique indicated that the significant relation between social media use and body dissatisfaction disappeared when the positive mother–adolescents relationship score was 4.08 or higher. Since only the interaction with positive mother–adolescent relationship was significant, H3 was partially supported.

### Gender Differences

To explore whether the associations of interest were different for boys and girls, two-way interactions between gender and positive father–adolescent and mother–adolescent relationships were added in Model 2 and three-way interactions between gender, social media use, and both positive parent–adolescent relationships were added in Model 3. No significant two-way or three-way interactions with gender appeared. Social media use and positive father–adolescent and mother–adolescent relationships were thus related to body dissatisfaction in similar ways among boys and girls. Likewise, the moderating role of positive father–adolescent and mother–adolescent relationships in the association between social media use and body dissatisfaction is equal for both genders.

## Discussion

Body dissatisfaction poses a significant threat to adolescents’ wellbeing (Stice and Bearman 2001). In order to combat body dissatisfaction, it is important to know what factors are associated with adolescents’ body dissatisfaction. Previous research has shown that adolescents who use social media more frequently report increased body dissatisfaction (Holland and Tiggemann 2016). However, the relationship between social media use and body dissatisfaction is stronger among some adolescents than others (Kleemans et al. 2018). To date, research on individual susceptibility to social media effects on body dissatisfaction has focused on individual traits, leaving the moderating role of social environmental factors under examined. The current study therefore investigated the moderating role of a social environmental factor: positive parent–adolescent relationships. More specifically, the current study tested if positive mother–adolescent and father–adolescent relationships attenuate the association between adolescent boys’ and girls’ social media use and their body dissatisfaction.

As hypothesized based on previous research (Holland and Tiggemann 2016) and the sociocultural model (Thompson et al. 1999), adolescents who used social media more frequently were more dissatisfied with their bodies in the current study. Similar to earlier studies (de Vries et al. 2016), gender did not moderate this relationship. It was also expected, based on previous studies (Bearman et al. 2006), that adolescents who report more positive relationships with their parents would report less body dissatisfaction. In addition, because adolescents who have more positive relationships are less susceptible to sociocultural influences on their body image (Cheng and Mallinckrodt 2009), it was hypothesized that the association between social media use and body dissatisfaction would be weakened by positive parent–adolescent relationships. However, the hypotheses concerning parent–adolescent relationships were only supported for mother–adolescent relationship and not for father–adolescent relationship. Among adolescents who reported more positive relationships with their mothers, the association between social media use and body dissatisfaction was weaker and these adolescents also reported lower body dissatisfaction overall. However, father–adolescent relationship was not connected to body dissatisfaction directly and did not moderate the association between social media use and body dissatisfaction. Gender did not moderate any of the relationships that were examined.

The findings of this study add to the body of research showing somewhat contrasting outcomes regarding the distinctive roles of mother–adolescent and father–adolescent relationships in adolescents’ body dissatisfaction and adolescent gender differences herein. In two previous studies, both positive aspects, namely intimacy and knowledge (May et al. 2006), and negative aspects, namely conflict (May et al. 2006) and communication difficulties (Al Sabbah et al. 2009), of the mother–adolescent relationship were only related to girls’ but not boys’ weight concern (May et al. 2006) and weight dissatisfaction (Al Sabbah et al. 2009). In the current study, positive mother–adolescent relationship was negatively related to body dissatisfaction among both boys and girls. So in contrast with previous research, the current study suggests that mother–adolescent relationships are not only related to the body image of daughters but also of sons.

In the present study, positive father–adolescent relationship was not related to girls’ or boys’ body dissatisfaction. At first glance this contradicts earlier research in which difficulties in communication with father were positively related to body weight dissatisfaction among both boys and girls (Al Sabbah et al. 2009) and conflict with father was associated with increased weight concern among both boys and girls (May et al. 2006). However, the present finding is in line with research that showed no relationship between father–adolescent knowledge and intimacy and girls’ and boys’ weight concern (May et al. 2006). Together, these findings tentatively suggest that, on average, positive aspects of the father–adolescent relationship (e.g., intimacy, knowledge) do not play a role in adolescent body image, but negative aspects of father–adolescent relationships might be related to the body image of both genders. However, the current study did not take negative aspects of parent–adolescent relationships into account as it focused specifically on potential beneficial relationships. Also, it is difficult to make sound comparisons between these studies as both the aspects of parent–adolescent relationships and of body image that were examined differ between the studies. Therefore, further research is needed to fully understand the role different positive and negative aspects of mother–adolescent and father–adolescent relationships play in adolescent boys’ and girls’ body dissatisfaction and other aspects of body image.

An explanation for the discrepancies in the findings for fathers and mothers in the current study could be related to cultural factors. In the country where this study was conducted, mothers are generally more involved in the lives of their sons and daughters than fathers are. Mothers tend to work less and spend more time with their children than fathers do in the Netherlands. Furthermore, if their parents are divorced, children are more likely to live with their mothers. Potentially as a result, mothers may influence the body dissatisfaction of their sons and daughters more than fathers do. This may be different in other cultures, and could explain the different findings across studies in different countries (Al Sabbah et al. 2009).

### Theoretical and Practical Implications

The current study advances knowledge about how different social environmental factors interact in their relationships with body dissatisfaction and increases the understanding of how and why media affect different individuals in dissimilar ways. The finding that positive mother–adolescent relationship moderated the association between social media use and body dissatisfaction is in line with the differential susceptibility approach to media effects (Valkenburg and Peter 2013). As also shown in previous research, the effects of social media differ from person to person and depend on how individuals process their social media experiences (de Vries et al. 2018). Moreover, the results extend knowledge about individual susceptibility to social media effects on body image by showing that not only individual traits but also social environmental factors, such as mother–adolescent relationship, can moderate the associations between social media use and body dissatisfaction. This is in line with the ecological approach to youth development (Bronfenbrenner and Morris 1998) and the role of media herein (Jordan 2004) and converges with findings in other areas of media effects research (Fikkers et al. 2013).

The current findings support the idea that positive relationships can protect adolescents against the detrimental effects of sociocultural messages about appearance on their body image (Cheng and Mallinckrodt 2009). If longitudinal and experimental research confirm that positive social relationships can indeed have such protective effects, efforts to improve adolescents’ relationships may have a desirable impact on their body dissatisfaction. Randomized control trials have shown that family-based approaches are effective in the treatment of body image related problems (Lock et al. 2010). Based on the current findings, interventions aimed at the family environment, particularly adolescents’ relationships with their mothers, may also have preventive effects by reducing the risk that appearance messages on social media increase adolescents’ body dissatisfaction. However, further research is needed before such recommendations can be made confidently.

### Limitations and Suggestions for Future Research

The current study is subject to some limitations that future research can improve on. First, the cross-sectional nature of the current study prevents drawing any conclusions about the direction of the effects. However, longitudinal research has indicated that social media use predicts body dissatisfaction over time and not vice-versa (de Vries et al. 2016). Furthermore, experimental research has shown causal effects of social media activities on adolescents’ body dissatisfaction (Kleemans et al. 2018). Although previous research supports the interpretation that it is the effect of social media on body dissatisfaction that is attenuated among adolescents with more positive mother–adolescent relationships, further experimental and prospective studies are needed to preclude other explanations.

Similar to the previous point, the current design cannot rule out alternative explanations and confounding factors regarding the relationships that were found. For example, BMI, an important predictor of body dissatisfaction (Holsen et al. 2012), was not measured in this study. Furthermore, other potential individual and social factors, such as level of puberty, peer and sibling influences, and mass media messages (Shroff and Thompson 2006), were not taken into account. Considering the small effect sizes found in the current study, many other factors likely also explain the differences in body dissatisfaction between adolescents. Future research should not only control for these influences but also investigate how different social and individual factors influence adolescents’ body dissatisfaction separately and in interaction with each other.

As the current sample is only from one (Western European) country and ethnically rather homogenous, the question is if the results generalize to samples from other countries and with other ethnicities. Similarly, adolescents with higher levels of education were overrepresented in the current study. Therefore, research in other countries and with more diversity in terms of ethnicity and education are needed to test if the findings of this study generalize to other populations.

More research is also needed to understand the mechanisms behind the attenuating role of positive mother–adolescent relationships. The current study indicates that a positive mother–adolescent relationship attenuates the deleterious connection between social media use and body dissatisfaction, but not why this is the case. Further research should therefore examine how adolescents who have a more positive relationship with their mother process social media experiences differently than adolescents who have a less positive mother–adolescent relationship. Studies may want to test the notion that adolescents with more positive mother–adolescent relationships indeed internalize and compare themselves less with appearance ideals on social media.

Another explanation worth investigating could be that a positive mother–adolescent relationship increases the quantity, quality, or the effectiveness of parental mediation regarding social media use. For example, research could test if mothers who have more positive relationships with their adolescent children are more likely to have conversations about the messages about physical appearance encountered on social media. Such forms of active parental mediation may protect adolescents against unwanted effects of social media on body dissatisfaction. Such research will not only further increase the theoretical understanding of (social) media effects on body dissatisfaction but also of the development of body dissatisfaction and of media effects more generally. Future research in this area should not exclusively focus on adolescent girls but also include boys in the investigation, as the findings applied to boys and girls to the same extent.

## Conclusion

Although on average social media use has undesirable effects on adolescents’ body dissatisfaction, not all adolescents are equally susceptible to this negative influence. The current study suggests that advantageous social environmental factors may attenuate the relationship between social media use and body dissatisfaction. More specifically, the relationship between social media use and body dissatisfaction was weaker among adolescent boys and girls who reported a more positive relationship with their mother. Furthermore, adolescents with more positive mother–adolescent relationships also reported less body dissatisfaction overall. Pending further experimental and longitudinal research, positive mother–adolescent relationships may thus have both a protective and a promotive impact on adolescents’ body image. If this is the case, improving the mother–adolescent relationship may be a promising way to combat body dissatisfaction and related problems. However, before such a recommendation can be confidently made, more research is needed to better understand if, how, and why positive and other aspects of parent–adolescent relationships impact adolescent body dissatisfaction and the effects of social media.
